# Weighing the evidence for pharmacological treatment interventions in mild COPD; a narrative perspective

**DOI:** 10.1186/s12931-019-1108-9

**Published:** 2019-07-08

**Authors:** Dave Singh, Anthony D. D’Urzo, James F. Donohue, Edward M. Kerwin

**Affiliations:** 1 University of Manchester, Medicines Evaluation Unit, Manchester University NHS Foundation Trust, Manchester, M23 9QZ UK; 2 Department of Family and Community Medicine, University of Toronto, Toronto, Ontario Canada; 3 Division of Pulmonary Diseases & Critical Care Medicine, University of North Carolina Pulmonary Critical Medicine, Chapel Hill, North Carolina, USA; 4 Clinical Research Institute, Medford, Oregon, USA

**Keywords:** Chronic obstructive pulmonary disease, Corticosteroid, Early intervention

## Abstract

There is increasing focus on understanding the nature of chronic obstructive pulmonary disease (COPD) during the earlier stages. Mild COPD (Global Initiative for Chronic Obstructive Lung Disease [GOLD] stage 1 or the now-withdrawn GOLD stage 0) represents an early stage of COPD that may progress to more severe disease. This review summarises the disease burden of patients with mild COPD and discusses the evidence for treatment intervention in this subgroup.

Overall, patients with mild COPD suffer a substantial disease burden that includes persistent or potentially debilitating symptoms, increased risk of exacerbations, increased healthcare utilisation, reduced exercise tolerance and physical activity, and a higher rate of lung function decline versus controls. However, the evidence for treatment efficacy in these patients is limited due to their frequent exclusion from clinical trials. Careful assessment of disease burden and the rate of disease progression in individual patients, rather than a reliance on spirometry data, may identify patients who could benefit from earlier treatment intervention.

**Electronic supplementary material:**

The online version of this article (10.1186/s12931-019-1108-9) contains supplementary material, which is available to authorized users.

## Background

Chronic obstructive pulmonary disease (COPD) is characterised by airflow obstruction that arises in response to exposure to noxious particles, commonly from cigarette smoking [1]. The current Global Initiative for Chronic Obstructive Lung Disease (GOLD) report recommends that following a spirometrically confirmed diagnosis of airflow limitation, an assessment based on a combination of exacerbation risk and symptom criteria is performed to evaluate severity and guide treatment decisions [1]. However, many patients remain undiagnosed until the more advanced stages of the disease [2, 3]. COPD is a heterogeneous condition, with a high degree of variation in the clinical presentation and rate of disease progression between individuals [4–6], and it has been suggested that greater emphasis should be placed on diagnosis and treatment earlier in the course of the disease to potentially slow progression [3, 7, 8].

Mild airflow limitation is defined by GOLD criteria as a post-bronchodilator forced expiratory volume in 1 s [FEV_1_]/forced vital capacity [FVC] ratio < 0.7 and a post-bronchodilator FEV_1_ ≥ 80% predicted (GOLD stage 1) [1]. The mean reported prevalence of GOLD stage 1 COPD ranges from 2.5% (European Community Respiratory Health Survey of adults aged 20–44 years in high-income countries) [9] to 8.1% (BOLD Study of adults aged ≥40) [10, 11]. Patients with GOLD stage 1 COPD frequently receive limited or no treatment [2]; however, these patients often suffer significant morbidity, including respiratory symptoms, exacerbations, limitation of exercise capacity and reduced physical activity [12, 13]. Some clinical trials suggest that patients with mild COPD may benefit from treatment intervention [14–16], but evidence is limited as most randomised controlled trials of the commonly used inhaled treatments have not enrolled patients with mild COPD [16, 17].

According to the classical Fletcher-Peto model [18], FEV_1_ decreases gradually over a lifetime in susceptible smokers, causing COPD. However, the rate of lung function decline in COPD is highly variable, being negatively affected by smoking and exacerbations, but also remaining relatively stable for long periods of time in many patients [5]. Furthermore, poor lung growth in early life – leading to low maximally attained FEV_1_ in early adulthood – also contributes to the development of COPD [4] and may lead to a COPD phenotype where airflow obstruction arises mainly due to poor lung growth. While some individuals with abnormal lung growth may suffer from accelerated lung function decline characteristic of COPD [18, 19] and go on to develop severe disease over time, other patients demonstrate a rate of lung function decline in later life that is lower compared with individuals who attained maximum lung growth [4]. Mild COPD therefore comprises a broad group of patients who have different disease trajectories, including many who have COPD mainly because of poor lung growth.

Early COPD has been defined broadly as ‘an interval in time at the beginning of the disease course’ [20]. A more precise operational definition of early COPD has recently been proposed for younger current or former smokers to identify individuals at high risk of rapid disease progression: patients aged < 50 years with ≥10 pack-years smoking history and one or more of the following: 1) post-bronchodilator FEV_1_/FVC < lower limit of normal; 2) compatible computed tomography (CT) abnormalities (visual emphysema, air trapping, or bronchial thickening graded mild or worse); 3) evidence of accelerated FEV_1_ decline (≥60 mL/year) that is accelerated relative to FVC [21]. It is important to recognise that ‘early’ and ‘mild’ COPD are different definitions, with the former focused on age of onset and the latter focused on pulmonary function [20]. Due to the challenges involved in the diagnosis of early COPD [20], there are currently few studies describing the clinical characteristics of this subgroup. The RETHINC study, which is currently ongoing, is a 12-week, Phase 3 study of the LABA/LAMA combination indacaterol/glycopyrrolate in symptomatic current and former smokers with normal FEV_1_ and may provide valuable insight into pharmacological intervention in patients with early COPD [22].

Mild COPD represents a group of patients who can practically be identified in clinical practice [11]. Mild COPD may progress to more severe and life-limiting disease over time in some individuals [11], although this progressive decline does not occur in all patients [20]. This has led to some doubts as to whether pharmacological treatment is required or if it would be effective in this group. Identification of patients with mild COPD with a greater disease burden and/or increased likelihood of disease progression may determine the most appropriate individuals for pharmacological interventions. Here, we provide a narrative review of the evidence concerning disease burden and progression in mild COPD. We also review the limited evidence to support pharmacological treatment intervention in this patient population. Given the sparse nature of clinical trial evidence to support pharmacological treatment interventions in mild COPD, we debate the optimum treatment approaches in this group.

### Search strategy

To evaluate different aspects of disease burden in mild COPD, we conducted PubMed searches for the terms ‘early COPD’, ‘mild COPD’, ‘GOLD stage 0’, ‘GOLD stage 1’, ‘emphysema AND normal lung function’ AND one of the following terms: ‘symptoms/dyspnoea/dyspnea/breathlessness/shortness of breath/chest tightness/cough/sputum/phlegm/wheeze’; ‘exacerbations’; ‘physical activity/exercise’ OR ‘FEV/FEV_1_/lung function decline/progression’. Descriptive reviews; commentaries; protocols; studies in a non-COPD therapy area (e.g. lung cancer, pulmonary hypertension, sleep apnoea); biomarker and genetic studies; diagnostic, methodological, prevalence/demographic, preference/adherence or comorbidity studies; studies not specifically in a mild COPD population/sub-population and studies without an informative comparator group were excluded unless of particular relevance.

### Disease burden in mild COPD

#### Symptom burden and health status

Respiratory symptoms associated with COPD, including breathlessness, cough, sputum and wheeze, have a profound impact on patients’ quality of life and overall health status [23–25]. Symptoms and their associated effects can precede the development of airflow limitation, as demonstrated in the SPIROMICS cohort [12], where respiratory symptoms were present in approximately 50% of current or former smokers with preserved FEV_1_/FVC ratio ≥ 0.7. The presence of respiratory symptoms in these subjects was associated with a higher rate of exacerbations and greater physical activity limitation versus asymptomatic subjects. It has also been reported that visual CT abnormalities were associated with higher COPD Assessment Test (CAT) scores in current or former smokers with normal lung function [26]. The importance of symptoms is illustrated by a study of patients with mild to moderate COPD (mean FEV_1_ 82.1% predicted); individuals with a CAT symptom score ≥ 10 had significantly greater work productivity loss than patients without COPD [27].

The literature search highlighted studies that assessed symptom burden specifically in patients with mild COPD (Table 1). In particular, the COPDGene study reported worse patient-reported outcomes and worse quality of life for mild COPD compared with controls; modified Medical Research Council (mMRC) score odds ratio 1.31, 95% confidence interval (CI) 1.10–1.56; St George’s Respiratory Questionnaire (SGRQ) score odds ratio 1.49, 95% CI 1.28–1.75 [28].

**Table 1:** Studies reporting symptom burden, health status, exacerbations and HCRU in patients with mild COPD^a^

Study	Population	Relevant outcome measure(s)	Finding in mild COPD versus controls
Symptom burden and health status studies			
Vaz Fragoso et al. 2016 [28]	Smokers with/without COPD (COPDGene cohort)	mMRC, SGRQ	Worse dyspnoea and HRQoL
Bridevaux et al. 2008 [29]	Never smokers or current and former smokers with COPD (SAPALDIA cohort)	SF-36	Worse HRQoL
Exacerbations and HCRU outcomes			
Dransfield et al. 2017 [30]	Smokers with/without COPD (COPDGene cohort)	Exacerbations, FEV_1_ decline	Exacerbations in mild COPD associated with greater FEV_1_ loss versus GOLD 0/2/3/4; exacerbation rate was similar for mild COPD versus GOLD 0 controls
Lee et al. 2016 [31]	COPD	Exacerbations	Lower exacerbation rate in mild COPD^b^ (0.4) versus GOLD 3/4 (0.9)
Garcia-Aymerich et al. 2011 [32]	Participants from CHS and ARIC cohorts with/without COPD	Hospitalisations due to COPD	Increased hospitalisation risk in mild COPD (adjusted IRR 2.1 and 3.2) versus controls
de Marco et al. 2004 [9]	Younger adults (20–44 years) from the ECRHS cohort with/without COPD	Patient-reported HCRU^c^	Greater HCRU in participants with COPD (all stages including stage 0) versus controls

Of the results identified by the search terms stated, only relevant, original studies including a mild or undiagnosed COPD population are shown. ARIC: Atherosclerosis Risk in Communities; CHS: Cardiovascular Health Study; COPD: chronic obstructive pulmonary disease; ECRHS: European Community Respiratory Health Survey; FEV_1_: forced expiratory volume in 1 s; GOLD: Global Initiative for Chronic Obstructive Lung Disease; HCRU: healthcare resource utilisation; HLQ: health and labour questionnaire; HRQoL: health-related quality of life; IRR: incidence rate ratio; mMRC: modified Medical Research Council Dyspnea Scale; SF-36: 36-item Short-Form Survey; SGRQ: St George’s Respiratory Questionnaire; SAPALDIA: Swiss Study on Air Pollution and Lung Diseases in Adults. ^a^mild COPD defined as GOLD 0 and/or 1 COPD, unless otherwise stated.^b^mild COPD defined as GOLD stage 1 and 2. ^c^including medication use, doctor visits and hospitalisations due to COPD

Primary care screening programmes have identified a high symptom burden in newly diagnosed patients with mild and moderate COPD [33, 34]. This illustrates the existence of a population, including patients with mild COPD, who suffer from potentially debilitating respiratory symptoms prior to receiving a diagnosis and gaining access to treatment.

#### Exacerbations

The rate of COPD exacerbations is related primarily to the history of previous exacerbations, but also to the severity of airflow limitation [35, 36]. However, Woodruff et al. [12] reported an increased rate of exacerbations in symptomatic current or former smokers (0.27, standard deviation [SD] ±0.67) compared with asymptomatic current or former smokers (0.08, SD ±0.31) or never-smokers (0.03, SD ±0.21; *p* < 0.001 for both comparisons), suggesting a subpopulation with increased exacerbation risk in the early stages of COPD.

Of the studies we identified (Table 1), two reported that exacerbation risk is relatively low in mild COPD and increases with the degree of airflow limitation [31, 32]. However, studies that compared GOLD stage 0/1 patients with control subjects with normal lung function [9, 32] found a significantly higher rate of exacerbations or healthcare utilisation in the GOLD stage 0/1 patients (adjusted incidence rate ratios 2.1 and 3.2, respectively; by comparison, 8.0 and 25.5 for GOLD 2, and GOLD 3 or 4, respectively [32]). It has also been reported that the association between exacerbation rate and FEV_1_ decline was stronger for patients in GOLD stage 1 than for patients in any other stage [30]. This suggests that the effect of exacerbations on FEV_1_ decline may be particularly harmful in the earlier stages of COPD.

#### Exercise tolerance and physical activity

The term ‘physical activity’ refers to the daily level of physical activity (such as time spent walking or exercising), and is dependent on numerous factors including physiological, behavioural, social and environmental influences. In contrast, ‘exercise tolerance’ (also referred to as exercise performance or capacity) is the amount of exercise an individual is capable of, and is often assessed using laboratory exercise tests such as the 6-min walk test [37, 38]. In patients with COPD, impaired lung function and respiratory symptoms (especially dyspnoea) lead to reduced physical activity and decreased exercise tolerance [37]. Patients avoid exertional dyspnoea by becoming less active, and the resultant deconditioning aggravates the symptom [39]. Thus, COPD leads to a significant reduction in patients’ activity levels, which worsens with increasing severity [39–41]. Maintaining regular physical activity reduces rates of hospitalisation and both all-cause and respiratory-related mortality in patients with COPD [42]; therefore, disrupting the downward spiral of inactivity at any stage of COPD may have substantial therapeutic value.

Our literature search identified many studies reporting reduced exercise tolerance in patients with mild COPD (Table 2), often using cycle testing [28, 41, 44, 47, 48, 51]. A common reason for this phenomenon is dynamic lung hyperinflation, which leads to impaired ventilation and dyspnoea [54]. The studies (see Additional file 1: Table S1) also show that ventilatory inefficiency [43, 48, 49] and diminished oxygen transport [47, 50, 52] occur even in patients with mild airflow limitation. Studies measuring physical activity in mild COPD have been less common; however, Watz et al reported no significant difference in mean steps per day and a higher proportion of patients who were predominantly sedentary when comparing patients with mild COPD with controls [41]. Patients with GOLD stages 2, 3 and 4 had significantly lower mean steps per day compared with controls and ranged from predominantly sedentary (GOLD stage 2) to very inactive (GOLD stages 3 and 4) [41]. Overall, these studies demonstrate a reduction in physical activity and exercise capacity in mild COPD associated with abnormalities of gas exchange.

**Table 2:** Studies reporting physical activity and exercise capacity in patients with mild COPD^a^

Study	Population	Relevant outcome measure(s)	Finding
Jones et al. 2017 [43]	Mild to moderate COPD (post-bronchodilator FEV_1_ ≥ 60% predicted); controls without COPD	Incremental exercise test	Increased dyspnoea and ventilatory inefficiency in mild-to-moderate COPD versus controls
Caram et al. 2016 [44]	Never smokers; smokers with/without mild-to-moderate COPD (post-bronchodilator FEV_1_ > 50% predicted)	6MWT	Lower exercise capacity in mild-to-moderate COPD versus never smokers
Vaz Fragoso et al. 2016 [28]	Smokers with/without COPD (COPDGene cohort [45, 46])	6MWT	Lower exercise capacity in mild COPD versus controls (non-significant)
Elbehairy et al. 2015 [47]	Mild COPD; non-smoker controls	Symptom-limited cycle test	Gas exchange abnormalities with increased dyspnoea and exercise intolerance in mild COPD versus controls
Neder et al. 2015 [48]	COPD; controls without COPD	Symptom-limited cycle test	Increased ventilatory inefficiency and reduced exercise capacity in mild COPD versus controls
Guenette et al. 2014 [49]	Mild COPD; controls without COPD	Symptom-limited cycle test	Increased ventilatory requirements and respiratory effort during exercise in mild COPD versus controls
Chin et al. 2013 [50]	Mild COPD; controls without COPD	Symptom-limited cycle test	Reduced peak O_2_ uptake; no peak end-inspiratory lung volume increase in mild COPD versus controls
Díaz et al. 2013 [51]	Dyspnoeic (mMRC score ≥ 1) and non-dyspnoeic patients with mild COPD; smoker controls	Borg dyspnoea rating, 6MWT	Decreased inspiratory capacity and increased ventilatory demand during exercise and reduced exercise capacity in dyspnoeic COPD versus non-dyspnoeic COPD or controls
Watz et al. 2009 [41]	COPD; controls with chronic bronchitis	Steps/day, minutes of at least moderate activity, 6MWT	Higher proportion of sedentary patients in mild COPD versus chronic bronchitis
Ofir et al. 2008 [52]	Symptomatic current or former smokers with mild COPD; age- and sex-matched former or non-smoker controls	Symptom-limited cycle test, Borg dyspnoea rating	Increased ventilatory requirements and exertional dyspnoea, decreased peak O_2_ uptake in mild COPD versus controls
Carter et al. 1993 [53]	COPD (FEV_1_/FVC, 0.6–0.7; FEV_1_ ≥ 60% predicted); controls without COPD	Resting and peak exercise gas exchange (with symptom-limited cycle test)	Decreased maximal oxygen consumption and ventilation, reduced work capacity and maximal heart rate in COPD versus controls

A total of 59 results were identified by the search terms stated; only relevant, original studies including a mild or undiagnosed COPD population are shown. 6MWT: 6-min walk test; COPD: chronic obstructive pulmonary disease; FEV_1_: forced expiratory volume in 1 s; FVC: forced vital capacity; GOLD: Global Initiative for Chronic Obstructive Lung Disease; LLN: lower limit of normal; mMRC: modified Medical Research Council Dyspnoea Scale. ^a^mild COPD defined as GOLD 0 and/or 1 COPD, unless otherwise stated

#### Lung function decline and disease progression

Accelerated decline of lung function is a characteristic feature of COPD [18]; however, since COPD results from a complex interaction between genetic factors [55] and the environment (smoking, exposure to dust/gases, burning of solid fuels/biomass, socioeconomic status) and is heterogeneous in nature [1, 56], it is perhaps no surprise that the progression of the disease is also heterogeneous, with rates of decline reported to vary widely both between individuals and between studies [4–6]. In addition, it has been suggested that FEV_1_ decline is inversely correlated with GOLD severity stage, with more rapid decline reported in patients with mild and moderate COPD than in those with severe/very severe COPD [5, 30, 57].

**Fig. 1:**
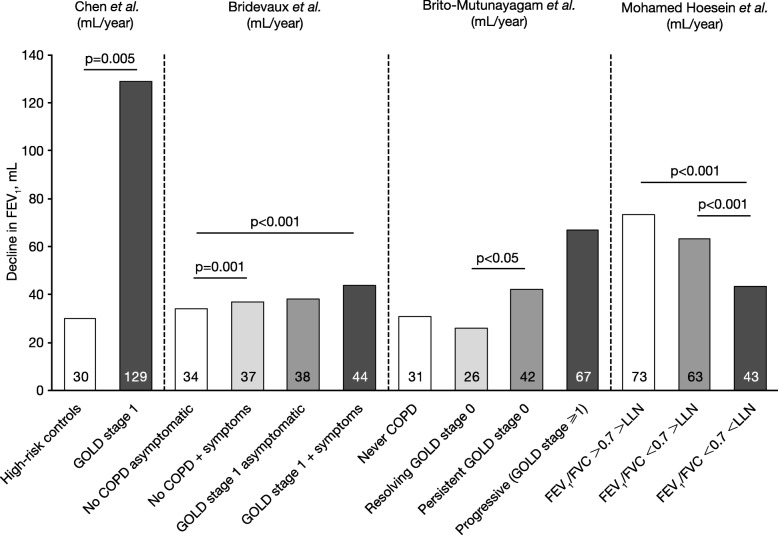
Studies reporting lung function decline in patients with mild COPD: Chen et al. [58], Bridevaux et al. [29], Brito-Mutunayagam et al. [59], Mohamed Hoesein et al. [60]. Inclusion criteria/study design: Chen et al. [58], subjects aged 45–80 years with a history of smoking or exposure to second-hand smoke for > 10 years; high-risk control group had post-bronchodilator FEV_1_/FVC > 0.7 and FEV_1_ < 95% predicted; mild COPD group had post-bronchodilator FEV_1_/FVC < 0.7 and FEV_1_ > 80% predicted in the absence of bronchodilator or inhaled corticosteroid; Bridevaux et al. [29], Swiss Study on Air Pollution and Lung Diseases in Adults cohort; considered symptomatic if chronic cough, phlegm or shortness of breath while walking reported at baseline (age range 18–60 years); Brito-Mutunayagam et al. [59], subjects aged ≥18 years from the North West Adelaide Health Study cohort; resolution, persistence or progression of GOLD stage 0 determined at 3.5-year follow-up; Mohamed Hoesin et al. [60], Dutch Belgian Lung Cancer Screening Trial; male heavy smokers (age range 47–80 years). Data shown are calculated from 3-year data described by Mohamed Hoesin et al. [60]. COPD: chronic obstructive pulmonary disease; FEV_1_: forced expiratory volume in 1 s; FVC: forced vital capacity; GOLD: Global Initiative for Chronic Obstructive Lung Disease; LLN: lower limit of normal

The literature search identified several papers reporting data for disease progression in patients with mild COPD or smokers with abnormal lung function (Fig. 1) [29, 58–60]. There is evidence that patients with mild COPD have an accelerated FEV_1_ decline compared with controls without COPD (Fig. 1) [29, 58]) and GOLD stages 2–4 [29], which is more prominent in patients with greater symptoms [29, 59]. In contrast, one study reported that male heavy smokers with FEV_1_/FVC > 0.7 had a more rapid rate of decline irrespective of whether they were above or below the lower limit of normal (LLN), compared with individuals with FEV_1_/FVC < 0.7 and < LLN, (Fig. 1) [60].

#### Mortality

**Fig. 2:**
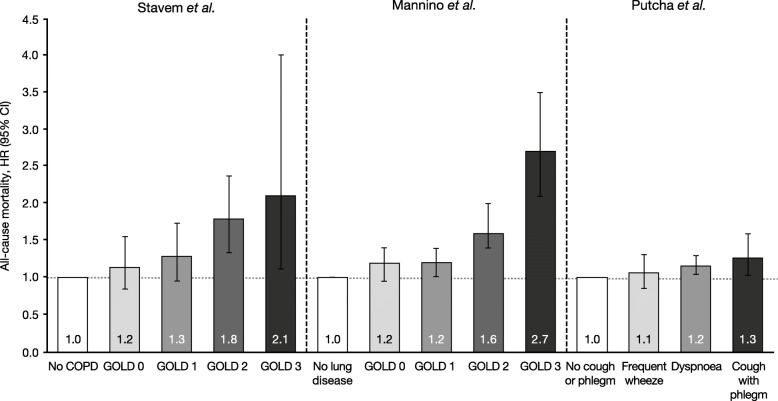
Studies reporting mortality in patients with mild COPD: Stavem et al. [61], Mannino et al. [62], Putcha et al. [63]. Inclusion criteria/study design: Stavem et al. [61], multivariate analysis of all-cause mortality over 26 years in an occupational cohort of men aged 40–59 years (data excluding all never-smokers), adjusted for age, smoking status, physical fitness, BMI, systolic blood pressure and serum cholesterol; Mannino et al. [62], multivariate analysis of all-cause mortality over 22 years in participants aged 25–74 years from the first National Health and Nutrition Examination Survey follow-up cohort, adjusted for lung function category, age, race, sex, education, smoking status, pack-years smoked, years since regularly smoked and BMI; Putcha et al. [63], all-cause mortality over 12.5 years in smokers aged 35–60 years with pre-bronchodilator FEV_1_/FVC < 0.7 and FEV_1_ 55–90% predicted from the Lung Health Study I and III cohorts, adjusted for age, gender, race, smoking status at Year 5, baseline FEV_1_, pack-years smoked and randomisation group. BMI: body mass index; CI: confidence interval; COPD: chronic obstructive pulmonary disease; FEV_1_: forced expiratory volume in 1 s; FVC: forced vital capacity; GOLD: Global Initiative for Chronic Obstructive Lung Disease; HR, hazard ratio

Our searches identified two studies that evaluated mortality risk in patients with mild COPD (Fig. 2) [61–63]. An analysis of all-cause mortality over 22 years in participants aged 27–74 from the First National Health and Nutrition Examination Survey follow-up cohort reported an increase in mortality risk in GOLD stage 1 (hazard ratio [HR] 1.2) compared with patients with no lung disease (HRs of 1.6 and 2.7 were calculated for GOLD stages 2 and 3, respectively) [62]. An analysis of all-cause mortality over 26 years in an occupational cohort of men aged 40–59 years (adjusting for patients who had never smoked) reported an increased mortality risk in GOLD stage 1 (HR 1.30) and symptomatic GOLD stage 0 (HR 1.35) compared with controls (HRs of 1.79 and 2.11 were calculated for GOLD stages 2 and 3, respectively) [61]. Additionally, in an analysis of all-cause mortality over 12.5 years in smokers aged 35–60 years with mild and moderate COPD, it was reported that the presence of cough and phlegm symptoms together was associated with increased mortality risk (HR 1.27); dyspnoea was also associated with higher mortality risk (HR 1.16) compared with no cough and phlegm symptoms [63]. Of note, a large proportion of deaths in patients with mild COPD have been found to be the result of cardiovascular complications [14, 64], with deaths due to respiratory disease becoming increasingly common as the severity of COPD increased [65]. This suggests that screening for cardiovascular comorbidities and/or preventative treatment intervention in patients with mild COPD may be of benefit.

### Effective interventions in mild COPD

In all patients with COPD, the elimination of risk factors through non-pharmacological interventions, such as smoking cessation, education, physical activity and (in some cases) pulmonary rehabilitation, forms an important component of the management strategy [1]. The GOLD report provides recommendations for pharmacotherapy and escalation/de-escalation strategies [1] based mainly on evidence from randomised controlled trials; however, the majority of such clinical trials have not included patients with mild COPD, so the evidence base for such recommendations in this population is less clear. Despite this, in the CanCOLD cohort of 5176 patients in primary care, 25% of patients diagnosed with mild COPD reported being prescribed pharmacological treatment for their COPD [66].

Our literature searches identified a range of interventional studies that assessed treatment effects in patients with mild COPD (pharmacological and non-pharmacological interventions summarised in Table 3 and Additional file 1: Table S1, respectively). However, it is important to note that the studies differed in the level of airflow limitation considered ‘mild’.

**Table 3:** Studies reporting pharmacological treatment efficacy in patients with mild COPD^a^

Study	Population	Intervention	Finding
Symptom burden			
Kanner et al. 1999 [67]	COPD (FEV_1_/FVC < 0.7 and FEV_1_ 55–90% predicted)	Smoking cessation intervention with SAMA (ipratropium bromide) or placebo, versus usual care	Lower prevalence of symptoms (no additional effect of SAMA). Presence of symptoms associated with greater FEV_1_ decline
Exacerbations			
Gartlehner et al. 2006 [68]	COPD, including mild COPD	ICS (budesonide, fluticasone, triamcinolone) versus placebo	Reduced exacerbation rate. Sub-analysis of 3 RCTs on mild COPD found no effect (*n* = 191)
Jones et al. 2003 [69]	COPD, stratified by severity	ICS (fluticasone propionate)	Reduced exacerbation rate in moderate/severe, but not mild COPD
Physical activity and exercise tolerance			
Hirai et al. 2017 [70]	Mild COPD (post-bronchodilator FEV_1_/FVC < 5th percentile LLN and FEV_1_ ≥ LLN)	Oral antioxidant (*N*-acetylcysteine) versus placebo	No effect on O_2_ transport or exercise tolerance
Gagnon et al. 2012 [71]	Mild COPD	SAMA/SABA (ipratropium bromide/salbutamol sulphate)	Improved FEV_1_ and hyperinflation; no significant increase in walking time
Lung function decline			
Zhou et al. 2017 [72]	Mild or moderate COPD	LAMA (tiotropium bromide) versus placebo	Improvement in pre- and post-dose FEV_1_; bronchodilator, reduced annual decline in post-dose FEV_1_
Wise et al. 2003 [73]	Smokers with mild COPD (FEV_1_/FVC < 0.7 and FEV_1_ 50–90% predicted)	SAMA (ipratropium bromide) versus placebo, both with smoking cessation intervention (plus a usual care control group)	No effect on airway responsiveness compared with placebo or usual care
Pauwels et al. 1999 [74]	COPD (pre-bronchodilator FEV_1_/FVC < 0.7 and post-bronchodilator FEV_1_ 50–100% predicted)	ICS (budesonide) versus placebo	Improvement in FEV_1_ decline after 6 months, but similar rate to placebo from 9 months to end of study (36 months)

COPD, chronic obstructive pulmonary disease; FEV_1_, forced expiratory volume in 1 s; FVC, forced vital capacity; ICS, inhaled corticosteroid; LAMA, long-acting muscarinic antagonist; LLN, lower limit of normal; RCT: randomised controlled trial; SABA: short-acting β_2_-agonist; SAMA: short-acting muscarinic antagonist. ^a^mild COPD defined as GOLD 0 and/or 1 COPD, unless otherwise stated

The efficacy of various pharmacological interventions in large trials where patients with mild COPD have been included has been reported, but often the number of patients in the mild COPD subgroup is limited (Table 3). While inhaled corticosteroid (ICS) treatment is effective in reducing exacerbations in moderate to severe COPD, the existing evidence in mild COPD is not sufficient to recommend ICS in these patients [68, 69]. Other studies investigating the effects of maintenance treatments including ICS and short-acting bronchodilators on lung function in patients with mild and moderate COPD have not shown any treatment effect [73, 74]. In contrast, a recent study reported a positive effect of tiotropium in reducing post-bronchodilator FEV_1_ decline between 0 and 24 months in patients with mild and moderate COPD, despite failing to meet its primary endpoint of reduced pre-bronchodilator FEV_1_ decline [72]. It should be noted, however, that no sub-analysis of mild COPD was presented in these studies, so the treatment effect in this group of interest is unclear. When studying patients with moderate COPD, tiotropium has been shown to improve lung function and patient-reported outcomes in patients who were naïve to maintenance therapy, suggesting benefits in initiating maintenance therapy early [75]; similar studies in mild COPD would be clinically informative. Studies specifically focused on mild COPD have not demonstrated any effect of either the oral antioxidant *N*-acetylcysteine or a short-acting muscarinic antagonist/short-acting β_2_-agonist combination on exercise tolerance [70, 71].

### Pharmacological intervention is warranted in mild COPD

Several studies have demonstrated a substantial disease burden in mild COPD compared with controls [9, 28–34]. Furthermore, there is extensive evidence of reduced physical activity and exercise capacity in mild COPD that is associated with abnormalities of gas exchange [28, 43, 47–49, 52, 53]. These findings demonstrate the presence of pathophysiological abnormalities in mild COPD associated with clinical consequences, and support the case to provide adequate bronchodilator treatment in these patients.

The examination of lung surgical specimens shows small airway destruction in mild COPD [76]. A recent study, also using surgical specimens, reported a decrease in the number of bronchioles in patients with GOLD stage 1 and GOLD stage 2 compared with control smokers, with a 40% reduction (*p* = 0.014) and a 43% reduction (*p* = 0.036) in the number of terminal bronchioles, respectively [77]. The remaining small airways showed features of narrowing and obstruction, while there was also a loss of alveolar surface area, with a 33% loss (*p* = 0.019) and a 45% loss (*p* = 0.002) in patients with GOLD stage 1 and GOLD stage 2, respectively [77]. These studies demonstrate the presence of significant pathology in mild COPD, particularly in the small airways.

The importance of small airway disease in mild COPD has been confirmed by CT scanning using parametric response mapping in the COPDGene cohort (*n* = 1508); small airway disease was the main cause of gas trapping in mild to moderate COPD, with emphysema becoming more important in severe and very severe COPD [78]. Furthermore, 81% of patients with GOLD stage 1 and 42% of patients with GOLD stage 0 had evidence of emphysema or functional small airways disease through CT scanning [79]. Functional small airways disease was also associated with FEV_1_ decline in patients with GOLD stage 0 [78]. Overall, the evidence from these pathology and imaging studies demonstrates the presence of significant small airway disease in mild COPD, with progression to emphysema being a process that occurs subsequent to small airway remodelling and destruction. These mechanistic insights highlight the potential to target airway disease in mild COPD.

While exacerbations are more common in patients with moderate to severe COPD, there is evidence that some patients with mild COPD also suffer from these events. Exacerbations are associated with a greater FEV_1_ decline [80], and there is evidence that they have the greatest impact on FEV_1_ decline in mild COPD [30]. While the rate of exacerbations is higher in moderate to severe COPD, it appears that they are also important events in mild COPD. Research into the causes and prevention of exacerbations in mild COPD is sparse, but would be valuable given the impact of these events. Abnormal mucin production has been observed in patients with COPD and smokers without airflow limitation [81]; novel approaches to the treatment of mild COPD in the future might target mucins and their role in exacerbations.

The GOLD report recommends that antibiotics can be used to treat exacerbations in patients with COPD, guided by sputum purulence [1, 56]; however, there has been little research on using antibiotics for exacerbations in mild COPD. One randomised placebo-controlled study of 310 patients with mild-to-moderate COPD found that treatment of exacerbations with amoxicillin/clavulanate was more effective than placebo and significantly prolonged the time to the next exacerbation compared with placebo [82].

The rate of FEV_1_ decline is increased in patients with mild COPD compared with controls without COPD and GOLD stages 2–4 [29]. Targeted pharmacological intervention in mild COPD could focus on specific patient subgroups, namely those with: (1) high symptom burden; (2) evidence of exacerbations; and (3) evidence of FEV_1_ decline. A long-acting bronchodilator should be the first-line treatment, as there is supporting evidence that these medicines can address symptoms, exacerbations and disease progression in patients with moderate COPD [72, 83–85], although we accept that direct evidence in mild COPD is lacking. Nevertheless, the advantage of the targeted approach proposed is to more intensively treat patients who are in greater need of symptomatic relief or who are at greater risk of disease progression.

### Challenges in implementing pharmacological intervention in mild COPD

Current pharmacological treatment recommendations, such as those of GOLD, are not based on lung function, but focus instead on the categorisation of patients according to symptoms and history of exacerbations [1]. While there is clearly a logical rationale to this approach, the magnitude of effects of pharmacological interventions may vary according to disease severity. The lack of evidence to support the use of common maintenance COPD treatments (namely long-acting muscarinic antagonists, long-acting β_2_-agonists, ICS and their combinations) in mild COPD is a concern. The optimum pharmacological treatment pathways for such patients remain unclear; properly designed clinical trials in patients with mild COPD are needed before any robust recommendations regarding pharmacological management can be made.

The problem with the simplistic approach of focusing on patients with GOLD stage 1 is that there are considerable differences in FEV_1_ trajectory that exist between individuals [4, 5]. Importantly, individuals who had impaired lung growth in early life, and thus begin their FEV_1_ decline from a lower starting point [4], may be classed as having mild COPD even though they may never progress to moderate or severe COPD. The focus on mild COPD could lead to unnecessary treatment (with the potential for adverse effects) for some individuals if intervention in mild COPD was adopted indiscriminately as a management strategy. Furthermore, there is the danger of prescribing inhaled treatments for symptoms that are caused by COPD comorbidities, such as cardiovascular disease.

Although longitudinal cohort studies have followed patient populations with COPD for over 20 years [4], to date no studies have comprehensively described the pathological mechanisms associated with more rapid disease progression. There is a need to improve our understanding of the disease mechanisms and inflammatory processes responsible for disease progression that could be targeted with pharmacological intervention. This includes susceptibility to bacterial infection, mucus hypersecretion and small airway remodelling [86]. Understanding the mechanisms responsible for small airways inflammation and remodelling that occurs before the clinical diagnosis of COPD may help to identify new targets for pharmacological intervention beyond those of commonly used bronchodilators and would provide an opportunity for earlier intervention; this has the potential to reduce airway remodelling earlier in the disease process and slow disease progression.

## Conclusions

Existing literature demonstrates that many patients with mild COPD suffer a substantial disease burden. While this suggests that patients with mild COPD could benefit from treatment intervention, the evidence for treatment efficacy in these patients is limited due to their exclusion from many clinical trials. We propose a practical solution in this situation, to target pharmacological management towards patients with mild COPD with greater symptoms, the presence of exacerbations and/or evidence of disease progression.

There is currently much interest in the concept of early COPD. However, identifying patients with mild COPD remains a relatively straightforward process, and offers the opportunity to identify patients at high risk of disease progression. Clinical trials of established and novel treatments are needed in this subgroup.

## Additional file

Additional file 1: **Table S1.** Description of data: Studies reporting non-pharmacological treatment efficacy in patients with mild COPD. (PDF 17 kb)

## Data Availability

Data sharing is not applicable to this article as no datasets were generated or analysed during the current study.

## Abbreviations

6MWT: 6-min walk test
ARIC: Atherosclerosis Risk in Communities
BMI: Body mass index;
CAT: COPD assessment test
CHS: Cardiovascular Health Study
CI: Confidence interval
COPD: Chronic obstructive pulmonary disease
CT: Computed tomography
ECRHS: European Community Respiratory Health Survey
FEV_1_: Forced expiratory volume in 1 s
FVC: Forced vital capacity
GOLD: Global Initiative for Chronic Obstructive Lung Disease
HCRU: Healthcare resource utilisation
HLQ: Health and labour questionnaire
HR: Hazard ratio
HRQoL: Health-related quality of life
ICS: Inhaled corticosteroid
IRR: Incidence rate ratio;
LABA: Long-acting β_2_-agonist
LAMA: Long-acting muscarinic antagonist
LLN: Lower limit of normal
mMRC: Modified Medical Research Council dyspnea scale
RCT: Randomised controlled trial
SABA: Short-acting β_2_-agonist;
SAMA: Short-acting muscarinic antagonist
SAPALDIA: Swiss Study on Air Pollution and Lung Diseases in Adults
SD: Standard deviation
SF-36: 36-item Short-Form Survey
SGRQ: St George’s Respiratory Questionnaire
