# Supplementary material for: Weighing the evidence for pharmacological treatment interventions in mild COPD; a narrative perspective
Source: Respir Res. 2019 Jul 8;20:141. doi: 10.1186/s12931-019-1108-9 (PMC6615221; doi:10.1186/s12931-019-1108-9)
Supplement: Supplementary file 1 — Table S1. Description of data: Studies reporting non-pharmacological treatment efficacy in patients with mild COPD. (PDF 17 kb) [file 12931_2019_1108_MOESM1_ESM.pdf]

Additional File 1

Supplementary Table 1. Studies reporting non-pharmacological treatment efficacy in patients with mild COPD#

Study	Population	Intervention	Finding
**Symptom burden**			
Jácome *et al*. 2014 [1]	Mild COPD	Pulmonary rehabilitation (exercise training and psychoeducation)	Improved dyspnoea, functional balance, muscle strength, exercise tolerance and SGRQ symptom, activity and total scores after 12 weeks
**Physical activity and exercise tolerance**			
Jácome *et al*. 2016 [2]	Mild or moderate/severe COPD	Pulmonary rehabilitation (exercise training and psychoeducation)	Improved 6MWT and physical activity in both mild and moderate/severe. Reduced exacerbations and improved SGRQ in both, greater in moderate/severe
Burtscher *et al*. 2009 [3]	Patients at risk for or with mild COPD	Intermittent hypoxia *versus* normoxia	Increased haemoglobin mass, total exercise time and exercise time to anaerobic threshold
Clark *et al*. 2000 [4]	With/without mild COPD	Weight training *versus* control	Improved muscle function and whole body endurance in mild COPD

Lung function decline

Kanner *et al*. 2001 [5]	Smokers with mild COPD (FEV_1/FVC <0.7 and FEV_1 55–90% predicted)	Smoking cessation intervention	Fewer lower respiratory illnesses in sustained quitters than continuing smokers. FEV_1 stable in sustained quitters, decreased in smokers.

6MWT: 6-minute walk test; COPD: chronic obstructive pulmonary disease; FEV_1: forced expiratory volume in 1 s; FVC: forced vital capacity; SGRQ: St George's Respiratory Questionnaire. [#]: mild COPD defined as GOLD 0 and/or 1 COPD, unless otherwise stated.

References

1 Jácome C, Marques A. Pulmonary rehabilitation for mild COPD: a systematic review. *Respir Care* 2014: 59: 588-594.

2 Jácome C, Marques A. Short- and long-term effects of pulmonary rehabilitation in patients with mild COPD: a comparison with patients with moderate to severe COPD. *J Cardiopulm Rehabil Prev* 2016: 36: 445-453.

3 Burtscher M, Haider T, Domej W, Linser T, Gatterer H, Faulhaber M, Pocecco E, Ehrenburg I, Tkatchuk E, Koch R, Bernardi L. Intermittent hypoxia increases exercise tolerance in patients at risk for or with mild COPD. *Respir Physiol Neurobiol* 2009: 165: 97-103.

4 Clark CJ, Cochrane LM, Mackay E, Paton B. Skeletal muscle strength and endurance in patients with mild COPD and the effects of weight training. *Eur Respir J* 2000: 15: 92-97.

5 Kanner RE, Anthonisen NR, Connett JE. Lower respiratory illnesses promote FEV_1 decline in current smokers but not ex-smokers with mild chronic obstructive pulmonary disease: Results from the Lung Health Study. *Am J Respir Crit Care Med* 2001: 164: 358-364.
